# The moss traits that rule cyanobacterial colonization

**DOI:** 10.1093/aob/mcab127

**Published:** 2021-10-10

**Authors:** Xin Liu, Kathrin Rousk

**Affiliations:** 1 CAS Key Laboratory of Mountain Ecological Restoration and Bioresource Utilization & Ecological Restoration and Biodiversity Conservation Key Laboratory of Sichuan Province, Chengdu Institute of Biology, Chinese Academy of Sciences, Chengdu 610041, China; 2 Department of Biology, Terrestrial Ecology Section, University of Copenhagen, Universitetsparken 15, 2100 Copenhagen, Denmark; 3 Center for Permafrost (CENPERM), University of Copenhagen, Øster Voldgade 10, 1350 Copenhagen, Denmark

**Keywords:** Bryophytes, moss colony, Cyanobacteria, nitrogen fixation, water retention, functional trait

## Abstract

**Background and Aims:**

Cyanobacteria associated with mosses represent a main nitrogen (N) source in pristine, high-latitude and -altitude ecosystems due to their ability to fix N_2_. However, despite progress made regarding moss–cyanobacteria associations, the factors driving the large interspecific variation in N_2_ fixation activity between moss species remain elusive. The aim of the study was to identify the traits of mosses that determine cyanobacterial colonization and thus N_2_ fixation activity.

**Methods:**

Four moss species varying in N_2_ fixation activity were used to assess cyanobacterial abundance and activity to correlate it with moss traits (morphological, chemical, water-balance traits) for each species.

**Key Results:**

Moss hydration rate was one of the pivotal traits, explaining 56 and 38 % of the variation in N_2_ fixation and cyanobacterial colonization, respectively, and was linked to morphological traits of the moss species. Higher abundance of cyanobacteria was found on shoots with smaller leaves, and with a high frequency of leaves. High phenol concentration inhibited N_2_ fixation but not colonization. These traits driving interspecific variation in cyanobacterial colonization, however, are also affected by the environment, and lead to intraspecific variation. Approximately 24 % of paraphyllia, filamentous appendages on *Hylocomium splendens* stems, were colonized by cyanobacteria.

**Conclusions:**

Our findings show that interspecific variations in moss traits drive differences in cyanobacterial colonization and thus, N_2_ fixation activity among moss species. The key traits identified here that control moss-associated N_2_ fixation and cyanobacterial colonization could lead to improved predictions of N_2_ fixation in different moss species as a function of their morphology.

## INTRODUCTION

Nitrogen (N) fixation performed by moss-associated cyanobacteria is a main N source in many pristine ecosystems, such as boreal forests and subarctic tundra, where N deposition is low and plant growth is commonly limited by N availability (LeBauer and Treseder, 2008; Wang *et al*., 2010). Given the high abundance of mosses in these ecosystems, the association contributes significantly to ecosystems’ N cycle by accounting for up to 50 % of the total N input (e.g. Rousk and Michelsen, 2017). Wide-ranging taxa of mosses have been found to host cyanobacteria, but N_2_ fixation activity of the association varies greatly among moss species (Rousk *et al*., 2015; Stuart *et al*., 2021). For instance, N_2_ fixation activity in *Hylocomium splendens* can be more than double the activity found in *Pleurozium schreberi*, although they are co-dominant in boreal forests (Stuart *et al*., 2021), while the activity in *H. splendens* is only one-sixth the activity found in *Sphagnum* in arctic tundra (Rousk *et al*., 2015). To date, although many factors, such as nutrient availability (Gundale *et al*., 2011; Rousk *et al*., 2017) and moisture content (Rousk *et al*., 2015, 2018) affect N_2_ fixation in mosses, we do not know why these large variations in N_2_ fixation activity between mosses growing in the same habitat occur. This lack of understanding of the seemingly random colonization patterns among moss species hampers efforts to upscale N_2_ fixation across moss species at a larger scale.

Cyanobacteria are the dominant N_2_ fixer associated with mosses (Leppänen *et al*., 2013), and N_2_ fixation activity is commonly correlated with the number of epiphytic cyanobacterial cells on mosses (Rousk *et al*., 2013*a*). Thus, differences in the abundance of epiphytic cyanobacteria likely drive the interspecific variation of moss-associated N_2_ fixation rates. Cyanobacterial abundance, on the other hand, is affected by different factors, such as the capability to move or disperse, and the environmental factors affecting these processes (Solheim and Zielke, 2002). Among these factors, traits of the moss host, which provide microsites for epiphytic cyanobacteria (Dalton and Chatfield, 1985), might play a key role as moss traits vary greatly among species (Niinemets and Tobias, 2019), and certain moss species seem to be especially colonized by cyanobacteria (Solheim *et al*., 1996). However, to date, it remains unknown whether moss traits affect the colonization of cyanobacteria and, if so, which suite of moss traits facilitates cyanobacterial colonization that leads to differences between moss hosts. The paucity of data on the effects of species-specific moss traits on cyanobacterial colonization limits our understanding of the relationship between moss and cyanobacteria, and thereby of the functioning of moss-dominated ecosystems such as boreal forests.

Studies have repeatedly found that moisture promotes N_2_ fixation in moss–cyanobacteria associations (Smith, 1984; Jackson *et al*., 2011; Gundale *et al*., 2012; Rousk *et al*., 2018). This could be the result of either a direct positive effect of water availability on cyanobacterial activity, or indirectly, promoting the colonization of cyanobacteria, or a combination of both. To form an effective N_2_-fixing association with plants, vegetative cyanobacterial cells differentiate into motile and short-lived hormogonia with a gliding activity for 48–72 h (Meeks and Elhai, 2002). The capacity to move would affect the number of cyanobacteria colonizing mosses (Santi *et al*., 2013). A moist surface or liquid is required for hormogonia to glide or swim (Brahamsha and Bhaya, 2014), and it is likely that passive water flow carries cyanobacteria from lower parts of a moss shoot to its upper segments (Broady, 1979). Studies concerning the mobility of hormogonia are mainly focused on structures of cyanobacteria (e.g. Adams and Duggan, 2008; Wilde and Mullineaux, 2015), while the characteristics of the moss host may affect movement capacity and hence the abundance of epiphytic cyanobacteria. If moss colonies absorb water at different rates, the rate of water flow may result in differences in cyanobacterial abundance between moss species and thereby differences in N_2_ fixation.

Water retention capacity and water absorption rate of mosses may be important for hosting cyanobacteria, and are affected by colony structure (e.g. density and height) as well as by traits of individual shoots (e.g. leaf frequency). Indeed, moss colony density and height have been reported to correlate positively with water retention capacity (Proctor, 1982; Elumeeva *et al*., 2011), shoot morphology controls dehydration rate of mosses (Cruz de Carvalho *et al*., 2019), and leaf width is positively related to water retention in *Sphagnum* mosses (Bengtsson *et al*., 2020). However, to our knowledge, there is no empirical evidence that these hydrology-related traits affect cyanobacterial colonization of mosses. Moreover, moss traits might affect colonization rates directly. Mosses are assumed to host and protect cyanobacteria between or on their leaves (Dalton and Chatfield, 1985), which vary 54-fold in size and 28-fold in frequency among species (Niinemets and Tobias, 2019), and cyanobacterial filaments are found between moss stems and leaves (Solheim and Zielke, 2002). Thus, moss species with larger leaves and higher leaf frequency should host more cyanobacteria due to higher availability of colonization sites. Yet again, the relationships between colony- (e.g. frequency of shoots and colony height), shoot- (e.g. frequency of leaves and shoot length) and leaf-level (e.g. leaf width and area) traits and cyanobacteria colonization remain elusive.

Another possible mechanism that regulates cyanobacterial colonization is the production of chemical compounds by the host. Mosses are known to produce and accumulate inhibitory compounds like phenols (Erickson and Miksche, 1974). These compounds can lead to inhibition of bacterial growth (Rousk *et al*., 2013*a*) and contributes to the low decomposability of moss litter (Lang *et al*., 2009). Differences in phenol concentration of host mosses might lead to variation in cyanobacterial colonization and activity. Similarly, pH is an influential factor structuring microbial communities (Rousk *et al*., 2009) and affecting N_2_ fixation rates in mosses (Alvarenga and Rousk, 2021). To date, it is unknown if differences in the chemical environment among moss species lead to variations in the number of epiphytic cyanobacteria.

The purpose of the study reported herein was to identify the moss traits that affect cyanobacterial colonization, and thus N_2_ fixation activity, using four different moss species that have been shown to vary in N_2_ fixation. To accomplish this, we measured acetylene reduction rate as a measure of N_2_ fixation activity, and assessed cyanobacterial colonization and abundance at shoot and colony (group of shoots) level and linked this to water balance traits (maximum water content, water absorption rate and water loss rate of moss colonies), chemical traits (pH, total phenols), colony structural traits (frequency of shoots and height) and morphological traits (shoot length, frequency of leaves, leaf area, etc., Table 1) at shoot as well as at leaf level of the four moss species collected in the subarctic region. We hypothesized that (1) N_2_ fixation activity is correlated with cyanobacterial colonization in all investigated moss species, (2) the moss species that has the highest water absorption rate hosts the most cyanobacteria, (3) moss traits (e.g. frequency of shoots) that facilitate water absorption increase cyanobacterial colonization, (4) moss shoots with higher frequency of leaves host more cyanobacterial colonizers, and (5) high phenol concentration and low pH inhibit cyanobacterial colonization.

**Table 1.** Evaluated variables and their symbols, definitions and units

Symbol	Description	Unit
AR	Acetylene reduction rate	nmol g^−1^ DW h^−1^
F_L,Colonized_	Frequency of cyanobacteria colonized leaves	%
CC_L,St_	Cyanobacteria count on stem leaves	Cells leaf^−1^
CC_L,Br_	Cyanobacteria count on branch leaves	Cells leaf^−1^
CD_L,St_	Cyanobacteria density on stem leaves	Cells mm^−2^ leaf
CD_L,Br_	Cyanobacteria density on branch leaves	Cells mm^−2^ leaf
WC_Max_	Maximum water content	%
W_Absorb_	Time needed for moss colony to absorb water from air dried status to 50 % of maximum water content. Larger number means lower hydration rate.	min
W_Lose_	Time needed for moss colony to lose water from 100 to 50 % of maximum water content. Larger number means lower desiccation rate.	h
H_Colony_	Height of moss colony	mm
F_Sh_	Frequency of Shoot	Shoots cm^−2^
pH	pH	-
Phenol	Total phenol concentration	mg GAE g^−1^
[C]	Carbon concentration	%
[N]	Nitrogen concentration	%
L_Sh_	Length of shoot	mm
F_L_	Frequency of leaves – number of (stem and branch) leaves per unit shoot length	Leaves mm^−1^ shoot
W_L,Base,St_	Basal width of stem leaf	mm
W_L,Max,St_	Maximum width of stem leaf	mm
L_L,St_	Length of stem leaf	mm
A_L,St_	Area of stem leaf	mm^2^
W_L,Base,Br_	Basal width of branch leaf	mm
W_L,Max,Br_	Maximum width of branch leaf	mm
L_L,Br_	Length of branch leaf	mm
A_L,Br_	Area of branch leaf	mm^2^

## MATERIALS AND METHODS

### Moss sampling

Moss samples were collected at two sites in Northern Sweden. *Aulacomnium turgidum* (Wahlenb.) Schwägr., *Hylocomium splendens* (Hedw.) Schimp. and *Tomentypnum nitens* (Hedw.) Loeske (Supplementary Data Figure S1) were collected in June 2019 in a subarctic dry heath close to the Abisko Scientific Research Station (68°19′02″N, 18°50′04″E). The mean annual air temperature in Abisko is 0.2 °C, and the mean annual precipitation is 337 mm (30-year mean 1986–2015, Abisko Scientific Research Station 2016). The site was dominated by mosses and *Vaccinium uliginosum*, *Andromeda polifolia* and *Rhododendron lapponicum* (see Rousk and Michelsen, 2017). *Pleurozium schreberi* (Brid.) Mitt., which did not occur at this site, was collected in August 2019 in a boreal forest near Arvidsjaur (64°58′49″N 19°33′59″E). This extended the study to another species that has been shown previously to differ in N_2_ fixation rates compared to *H. splendens* despite their similar morphology and habitat preferences (Jean *et al*., 2020). Mean annual temperature and precipitation are approximately 1 °C and 570 mm respectively. The forest was dominated by *Picea abies*, *V. vitis-idaea*, *V. myrtillus* and *Empetrum hermaphroditum* (see Rousk *et al*., 2013*b*). From this site, samples of *H. splendens* were also collected in order to identify differences in the measured variables across ecosystems, as well as to ascertain if differences in N_2_ fixation is a species or an ecosystem effect.

Three separate monospecific moss colonies, with at least 5 m distance from each other, were selected for each species at the subarctic and boreal sites. Uniform moss colonies of 15 cm × 15 cm were sampled and carefully transported to the laboratory in Copenhagen. The samples were assessed for N_2_ fixation activity, water balance (maximum water content, water absorption and -loss rate), chemical (pH and total phenols), shoot and leaf morphological traits, and cyanobacterial counts. The *H*. *splendens* samples from the boreal forest site were assessed for N_2_ fixation activity, water balance, and chemical traits.

### Morphological traits and cyanobacterial colonization

Three shoots from each sample were randomly selected to measure shoot and leaf morphological traits (n = 9 per species). We measured the length of each shoot after submerging in double distilled (dd) H_2_O to ensure full hydration. For *H. splendens*, shoots were divided into three segments according to innate growth markers. These segments were: topmost current year segment which are younger than one-year (ca. 11 mm), the segment younger than 2 years but older than 1 year, and the segment older than 2 years. The stem and six random branches were chosen from each segment for leaf measurements and cyanobacterial counting. For *P. schreberi* and *T. nitens*, six random branches were chosen from each shoot. For *A. turgidum*, which does not have branches, only stems leaves were measured. Stems of *A. turgidum*, *P. schreberi* and *T. nitens* were divided into two sections: the top sections, approximately 9 mm for *A. turgidum*, 13 mm for *P. schreberi* and 8 mm for *T. nitens*, which represents 1 year’s length growth (Bauer *et al*., 2007), and other sections. Brown segments that were partly decomposed were not included in the measurements. Fully hydrated leaves from these chosen branches and stem sections (351 branches/stem sections in total: 18 for *A. turgidum*, 72 for *P. schreberi*, 72 for *T. nitens* and 189 for *H. splendens*) were measured.

To be able to link morphological traits to cyanobacterial colonization, we destructively harvested all the leaves from 1- to 3-mm lengths of stem segments or branches through scraping to enable counting. The number of scraped-off leaves was counted using an Olympus SZX16 stereo microscope. In total, 4857 leaves (328 for *A. turgidum*, 1141 for *P. schreberi*, 1469 for *T. nitens* and 1919 for *H. splendens*) were scraped off. The number of cyanobacteria-colonized leaves among scraped-off leaves was counted using an Olympus BX61 ultraviolet-fluorescence microscope with a green filter. The frequency of colonized leaves for each stem segment or branch was calculated according to the number of colonized leaves and the number of all scraped-off leaves. Digital images of five randomly selected leaves were taken with a USB2.0 CMOS Camera (ToupTek, Hangzhou, China) attached to the stereo microscope. From these images, the maximum width, basal width, length and area of individual leaves (Table 1) were measured with ImageJ 2.35 (Wayne Rasbund, National Institutes of Health, Bethesda, MD, USA). The cyanobacterial cells on the five selected leaves per stem segment or branch were counted using the Olympus BX61 ultraviolet-fluorescence microscope. The leaf size and number of cyanobacterial cells for 1755 leaves (90 for *A. turgidum*, 360 for *P. schreberi*, 360 for *T. nitens* and 945 for *H. splendens*) were measured. Microscopic counting, rather than using proxies of cyanobacterial quantity (Renaudin *et al*., 2021), enabled us to link leaf traits with cyanobacterial colonization.

### Water balance

#### Water absorption.

To measure water absorption rates of moss colonies, the bottom of transparent polypropylene cups, 3.7 cm in diameter, were cut off and replaced with cotton mesh (Supplementary Data Fig. S2). We placed a round moss colony, which fits the area of the cup, into the cup. Moss patches had similar height to those in the field, and included both green and basal parts, while decomposed parts were excluded.

Cups filled with moss colonies were air-dried in a ventilated growth chamber for one week. The air temperature in the chamber was 6 °C in the night (1800–0600 h) and 12 °C during daytime (0600–1800 h). The relative humidity in the chamber varied between 51.2 and 97.7 %, with an average of 76.6 %. The cups were then weighed and placed into a plastic box, which was filled with ddH_2_O to 1 cm depth. The cups were weighed at time intervals of 2 min for the initial 20 min, then weighed at intervals of 5 min for the rest of the first hour, and then weighed every hour until their mass became nearly constant. We added ddH_2_O to the box during the experiment to maintain the same water level throughout the measurements.

*Water loss*. The same cups filled with moss colonies were used to determine the water loss rate of the moss colonies. Water (ddH_2_O) was added to the box to immerse the moss colonies and left for 12 h, allowing the colonies to reach full hydration. The cups were then placed on a tilted plastic surface for 5 min to let the surplus water run down. The samples were weighed and kept in the same chamber as above and allowed to dry. The cups were weighed every hour during the initial 12 h and every 6 h for a maximum of 120 h until their mass became nearly constant. Then we counted the number of moss shoots in each cup, oven-dried the mosses at 65 °C for 48 h and recorded their dry weight.

*Calculations.* Maximum water content of moss colonies were calculated and expressed as percentage of dry weight (Table 1). Exponential functions weight = *K*/(1 + exp(*a* + *b* × time)) and weight = *a* × exp(−**b** × time) were fitted to the moss weight and time data in the water absorption and loss experiment, respectively. The parameters, *K*, *a* and *b*, were calculated using the “nls()” function in R. The exponential functions closely imitate the moss weight changes through time during water absorption and loss processes (Supplementary Data Fig. S2). The mean *R*^2^ of the water absorption relationship was 0.87 (0.76–0.94), and the mean *R*^2^ of the water loss relationship was 0.96 (0.87–0.99). The time for 50 % water absorption and the time for 50 % water loss, which is referred to later to hydration rate and desiccation rate, respectively, were calculated using “uniroot()” function in R (R Core Team, 2019).

### N_2_ fixation

Fully hydrated mosses were kept in the above-mentioned chamber for 1 week before measurement to minimize potential variability of N_2_ fixation activity between sampling times. N_2_ fixation activity was assessed using the acetylene reduction assay (ARA). For this, 20-mL glass vials containing ten fully hydrated moss shoots (*n* = 3 for each species) were sealed and 10 % of the headspace was replaced with acetylene. The moss samples were incubated for 10 h at 12 °C, 10 h at 6 °C, then 4 h at 12 °C. Ethylene generated in the headspace by the cyanobacterial nitrogenase enzyme was measured by gas chromatography with a flame ionization detector using an automatic headspace sampler (Agilent, 8890 GC System, Agilent, Santa Clara, USA). The fresh moss shoots were oven-dried at 65 °C for 48 h and ground into fine powder, which was subsequently used for total carbon (TC), total nitrogen (TN) and phenol concentration measurement.

### Nutrient concentrations, total phenols and pH measurements

Carbon and N concentrations in moss tissue were assessed with a Vario Macro Cube Elemental Analyzer (Elementar, Germany). Total phenols were measured in moss tissue that had been ground into fine powder and then suspended in 10 mL ethanol. Samples were shaken for 120 min and then centrifuged at 3600 g for 10 min. The supernatant was analysed for phenols using the Folin–Ciocalteu reagent. The absorbance was measured at 725 nm using a spectrophotometer. The pH of mosses was measured in 3 g fresh moss tissue that was submerged in 15 mL ddH_2_O and then shaken for 60 min. The pH of the extracts was determined with a pH electrode.

### Statistical analyses

We first performed principal component analyses (PCAs) using acetylene reduction rate and cyanobacterial colonization variables, as well as the water balance, chemical and morphological traits to obtain an overview of the multidimensional cyanobacterial colonization and moss traits spectrum of variation. Because of close relationships of cyanobacterial count, density and morphological traits of branch leaves to those of stem leaves, only variables and traits of stem leaves were included in the analyses. The relationships between acetylene reduction rate and frequency of colonized leaves were tested with linear regression analyses. We used linear regressions to identify the main traits driving the variation in cyanobacterial colonization and N_2_ fixation rate: (1) relationships of water balance traits (maximum water content, hydration and desiccation rate) with cyanobacterial activity and colonization; (2) the effects of colony trait (frequency of shoots), shoot and leaf traits (shoot length and leaf width) on hydration rate and cyanobacterial colonization; (3) the relationships between cyanobacterial colonization and shoot as well as leaf traits (frequency of leaves, leaf length maximum and basal leaf width, and leaf area); and (4) the effects of chemical traits (pH and phenols) on N_2_ fixation activity and cyanobacterial colonization.

Differences in N_2_ fixation activity across moss species were compared with one-way ANOVA. Species-specific differences in cyanobacterial colonization were compared by linear mixed models, in which species identity was included as a fixed effect and colony and shoot were included as random effects. The ANOVA and linear mixed models were followed by Tukey’s HSD test. Variation in frequency of colonized leaves across different sections of moss shoots as well as across moss species was compared by two-way ANOVA. Differences in N_2_ fixation activity, colony structure and chemical traits of *H. splendens* between sites were tested with *t*-tests. To demonstrate the variance pattern of moss traits, intra- and inter-specific coefficients of variation (CVs) of water balance, colony, chemical, shoot and leaf morphological traits were calculated. The differences in CV between intra- and inter- species were compared by Krishnamoorthy and Lee’s modified signed-likelihood ratio test (Marwick and Krishnamoorthy, 2019). Acetylene reduction rate, leaf cyanobacterial count and cyanobacterial density were log10-transformed before all analyses. All analyses were conducted with R v.3.6.1 (R Core Team, 2019), and all tests were considered significant when *P* < 0.05.

## RESULTS

### Moss species differences in N_2_ fixation activity

Large variations were found in N_2_ fixation activity and cyanobacterial colonization between moss species (*F*_3,14_ = 12.10, *P* < 0.001, Table 2). The highest N_2_ fixation activity was found in *H. splendens* (113.00 ± 67.18 nmol g^−1^ DW h^−1^), while the lowest N_2_ fixation was found in *P. schreberi* (0.69 ± 0.23 nmol g^−1^ DW h^−1^). The N_2_ fixation activities of *T*. *nitens* and *A*. *turgidum* were 42.02 ± 30.12 and 12.19 ± 3.93 nmol g^−1^ DW h^−1^, respectively. Accordingly, a higher frequency of *H. splendens* leaves (54.19 ± 9.36 %) was colonized compared with *T*. *nitens*, *A*. *turgidum* and *P. schreberi* leaves (19.14 ± 5.24, 34.54 ± 6.25 and 18.19 ± 2.76 %, respectively).

**Table 2.** Mean values and s.e. (*n* = 3) of acetylene reduction rates (AR, nmol g^−1^ DW h^−1^), frequency of colonized leaves (F_L,Colozied_, %), cyanobacteria count on stem leaf (CC_L,St_, cells per leaf) and branch leaf (CC_L,Br_, cells per leaf) and cyanobacteria density on stem leaf (CD_L,St_, cells mm^−2^ leaf area) and branch leaf (CD_L,Br_, cells mm^−2^ leaf area), and their differences among species. Differences in AR between species were tested with one-way ANOVA. Differences in cyanobacterial colonization were tested with a linear mixed model in which species was included as a fixed factor and colony and shoot were included as random effects

	Aulacomnium turgidum	Hylocomium splendens	Pleurozium schreberi	Tomentypnum nitens	F	df	P
AR	12.19 (3.93)	113.00 (67.18)	0.69 (0.23)	42.02 (30.12)	12.10	3,14	<0.001
F_L,Colonized_	34.54 (6.25)	54.19 (9.36)	18.19 (2.76)	19.14 (5.24)	7.04	3,24	0.001
CC_L,St_	23.20 (5.39)	28.95 (11.67)	7.50 (1.64)	12.97 (6.32)	1.91	3,23	0.156
CC_L,Br_	–	26.63 (8.65)	4.99 (0.77)	7.73 (2.61)	2.97	2,15	0.082
CD_L,St_	14.72 (3.72)	85.51 (36.21)	7.12 (2.18)	10.05 (3.91)	6.49	3,23	0.002
CD_L,Br_	–	135.84 (45.03)	7.75 (1.40)	13.32 (4.54)	9.80	2,15	0.002

### Covariation of N_2_ fixation activity, cyanobacterial colonization and moss traits

**Fig. 1.**
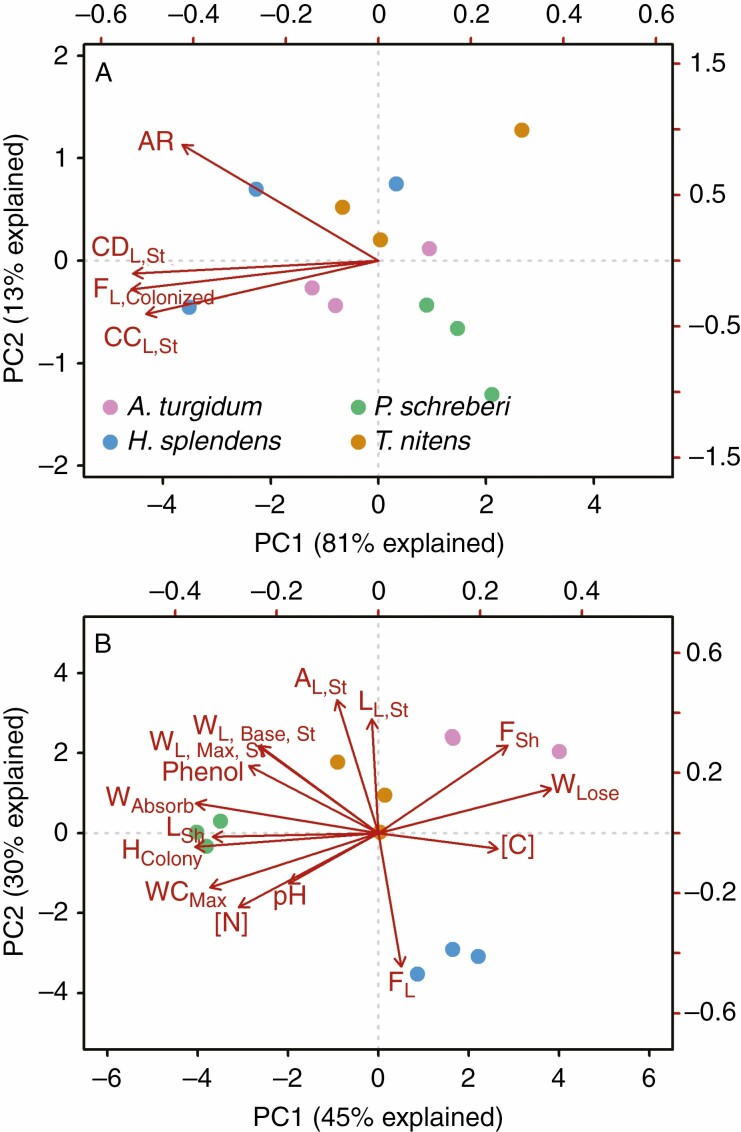
PCA of cyanobacterial colonization and acetylene reduction rate (A) and PCA of chemical and water balance, colony structural and shoot as well as leaf morphological traits (B). Acetylene reduction rate (AR), cyanobacteria count (CC) and cyanobacteria density (CD) were log10-transformed before analysis. Because of close relationships of cyanobacteria count, density and morphological traits of branch leaves to those of stem leaves, only variables and traits of stem leaves were included in the analyses.

**Fig. 2:**
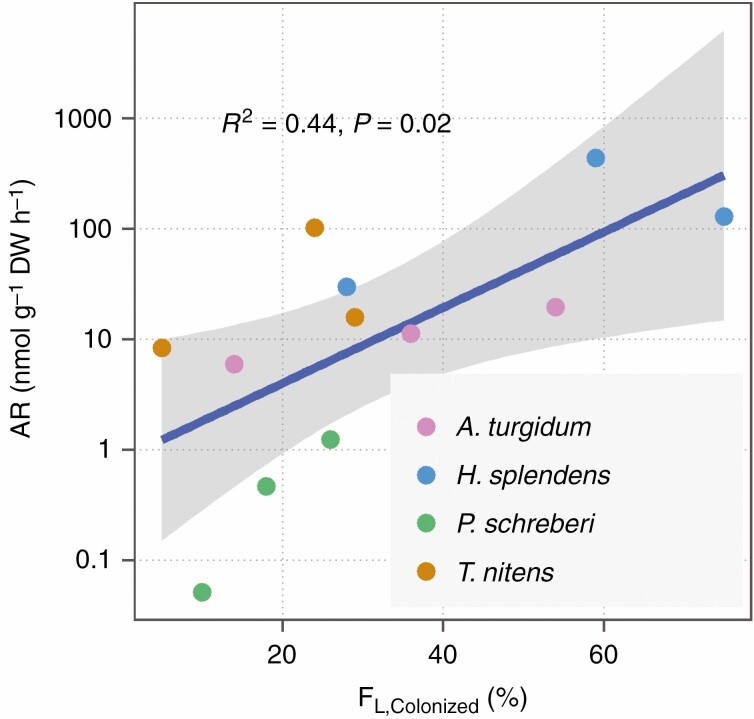
Acetylene reduction rate (AR) in relation to the frequency of leaves colonized by cyanobacteria (F_L,Colonized_). Each coloured dot represents one moss colony, which was grouped by species identity indicated with different colours. Acetylene reduction rate was log10-transformed before analysis. Frequency of colonized leaves is the mean value of three shoots (*n* = 3), and 10 (*A. turgidum*), 40 (*P. schreberi* and *T. nitens*) and 105 leaves (*H. splendens*) were examined on each shoot. The grey shading around the regression lines represents 95 % confidence intervals of the fitted values.

The PCA for N_2_ fixation activity and cyanobacterial colonization revealed one major principal component axis, which explained 81 % of the overall variance (Fig. 1A), suggesting a strong covariation of N_2_ fixation activity and cyanobacterial colonization. Confirming the patterns in the PCA, we found significant and positive correlations among N_2_ fixation activities and frequency of colonized leaves (F_L,Colonized_, Fig. 2), cyanobacterial count and density (CC_L,Br_, CD_L,St_ and CD_L,Br_, Supplementary Data Figure S3).

The PCA for moss traits revealed two major dimensions of moss traits covariation at colony level (Fig. 1B). The first PCA axis accounted for 45 % of the variation for moss traits and was mainly driven by water balance traits (hydration and desiccation rate) and shoot length. The second axis accounted for 30 % of the overall variance and was primarily related to leaf area and frequency of leaves, suggesting a leaf size versus leaf number trade-off (Supplementary Data Fig. S4).

### Cyanobacterial colonization and hydration rate

**Fig. 3:**
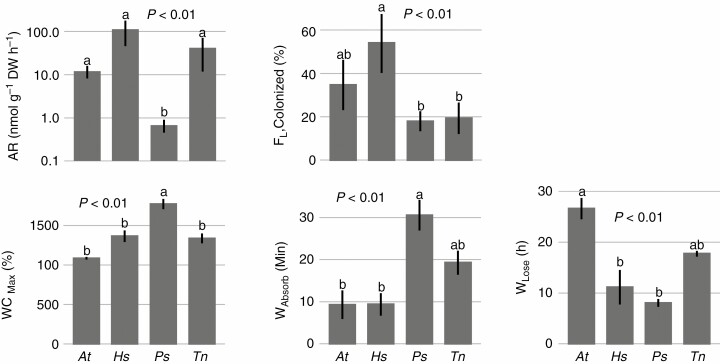
Differences in acetylene reduction (AR), frequency of colonized leaves (*F*_L,Colonized_), maximum water content (WC_Max_), time for 50 % water absorption (*W*_Absorb_, hydration rate) and time for 50 % water loss (*W*_Lose_, desiccation rate) among moss species. Larger *W*_Absorb_ and *W*_Lose_ indicate lower hydration rate and desiccation rate, respectively. Different lower case letters above error bars indicate significant (*P* < 0.05) differences among species according to Tukey’s HSD test. Letters on *x*-axes are acronyms of studied species, i.e. *At*, *Aulacomnium turgidum*; *Hs*, *Hylocomium splendens*; *Ps*, *Pleurozium schreberi*; *Tn*, *Tomentypnum nitens*.

Significant differences in frequency of colonized leaves (F_L,Colonized_, *F*_3,24_ = 7.03, *P* < 0.001) were found among species. Maximum water content (WC_Max_, *F*_3,14_ = 16.17, *P* < 0.001), hydration rate (time for 50 % water absorption, W_Absorb_, *F*_3,14_ = 10.27, *P* < 0.001) and decicassion rate (time for 50 % water loss, W_Lose_, *F*_3,14_ = 9.27, *P* = 0.001, Fig. 3) were significantly different among species. *Hylocomium splendens*, with the fastest hydration rate, had the highest cyanobacterial colonization frequency and N_2_ fixation activity.

**Fig. 4:**
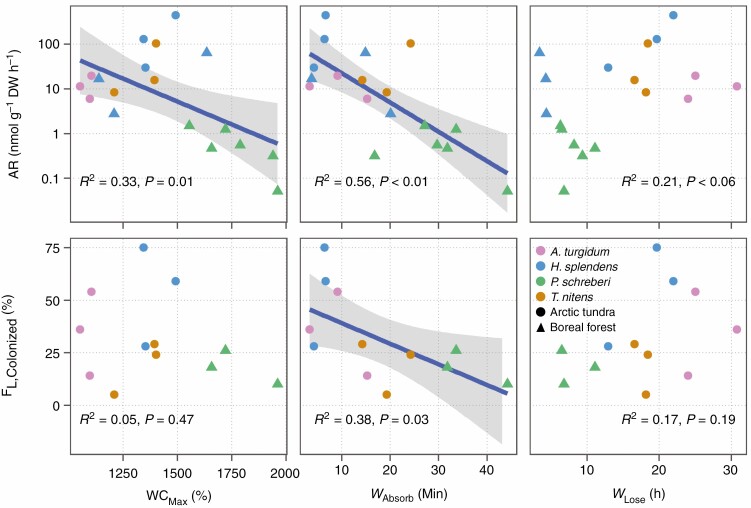
Acetylene reduction rate (AR, nmol g^−1^ dw h^−1^) and frequency of colonized leaves (*F*_L,Colonized_) in relation to water balance traits, maximum water content (WC_Max_), time for 50 % water absorption (*W*_Absorb_, hydration rate) and time for 50 % water loss (*W*_Lose_, desiccation rate). Each coloured dot represents one moss colony grouped by species identity indicated with different colours and ecosystem types, arctic tundra (filled circles) and boreal forest (filled triangles). The grey shading around the regression lines represent 95 % confidence intervals of the fitted values.

Regression analysis revealed a strong negative relationship between time for 50 % water absorption (W_Absorb_) and N_2_ fixation activity (i.e. positive relation between hydration rate and N_2_ fixation activity), and the moss colony hydration rate explained 56 % of the variation in N_2_ fixation activity (*R*^2^ = 0.56, *P* < 0.001, Fig. 4). A similar relationship was found between hydration rate and cyanobacterial colonization, for *W*_Absorb_ explained 38 % variation in frequency of colonized leaves (*F*_L,Colonized_, *R*^2^ = 0.38, *P* = 0.034, Fig. 4).

**Fig. 5:**
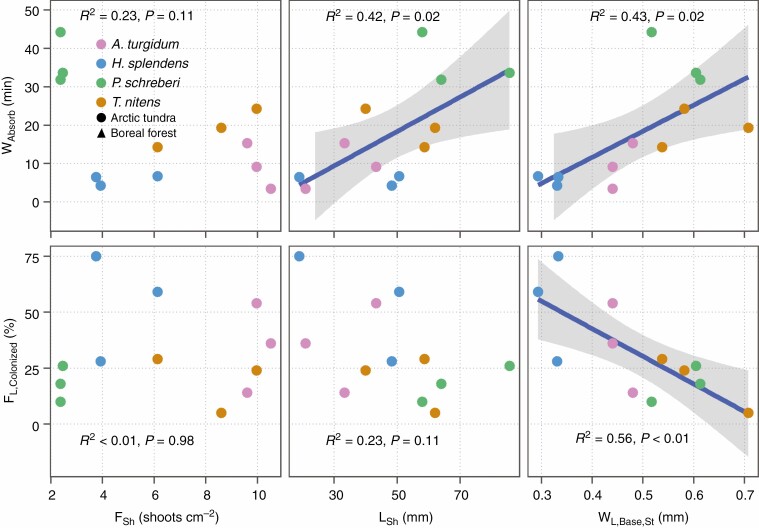
Time for 50 % water absorption (*W*_Absorb_, hydration rate) and frequency of colonized leaves (F_L,Colonized_) in relation to frequency of moss shoots (*F*_Sh_), shoot length (*L*_Sh_), and basal width of stem leaves (*W*_L,Base,St_). Each coloured dot represents one colony grouped by species identity indicated with different colours. *W*_Absorb_ and *F*_Sh_ were measured at colony level, and F_L,Colonized_, L_Sh_, and W_L,Base,St_ represent the mean value of three shoots. The grey shading around the regression lines represent 95 % confidence interval of the fitted values.

There was no significant relationship between shoot frequency (*F*_Sh_) and hydration rate. But shoot length (*L*_Sh_) and basal width of stem leaves (*W*_L,Base,St_) explained 42 % (*P* = 0.024) and 43 % (*P* = 0.020) variation in hydration rate (*W*_Absorb_), respectively (Fig. 5).

### Links between cyanobacterial colonization and morphological traits

The longer the moss shoot (*L*_Sh_), the fewer leaves were colonized at shoot level (*F*_L,Colonized_; *R*^2^ = 0.13, *P* = 0.033, Supplementary Data Fig. S5). Similarly, shoot length was negatively correlated with cyanobacterial count (branch leaves, *R*^*2*^ = 0.21, *P* = 0.020) and density at shoot level (stem leaves, *R*^2^ = 0.16, *P* = 0.018; branch leaves, *R*^2^ = 0.24, *P* = 0.011, Supplementary Data Fig. S5). A higher frequency of leaves was colonized in the lower sections (below 15 mm) than in the top section (first 11–15 mm) of the moss shoot in all four moss species (Supplementary Data Fig. S6).

**Fig. 6:**
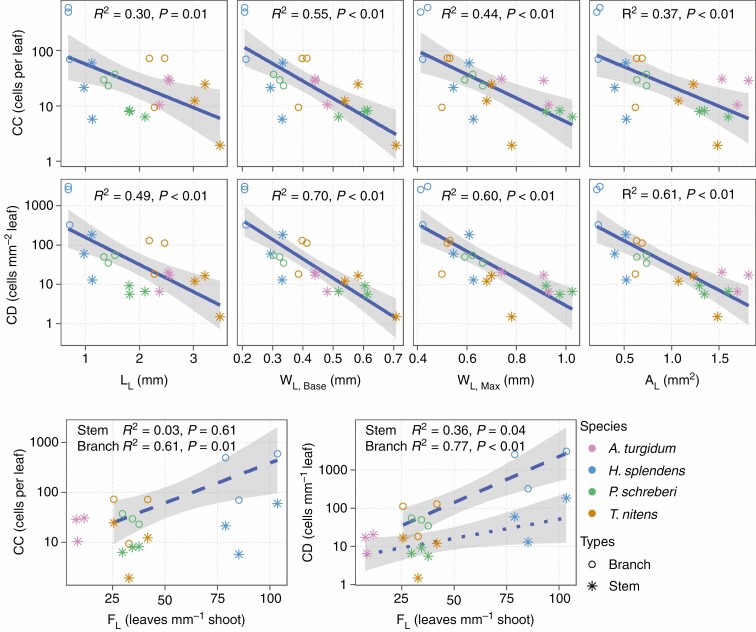
Cyanobacterial cells per leaf (CC) and per leaf area (CD) in relation to leaf morphological traits, leaf length (L_L_), basal width (W_L,Base_), maximum width (W_L,Max_) and leaf area (A_L_), as well as the cyanobacterial abundance (CC and CD) in relation to frequency of leaves (F_L_). Each coloured points represents mean value of one colony. The grey shading around the regression lines represent 95 % confidence interval on the fitted values.

Fewer leaves were colonized (*F*_L,Colonized_) with increasing leaf basal width (*W*_L,Base,St_, *R*^2^ = 0.56, *P* = 0.005, Fig. 5). Cyanobacterial count (CC) and density (CD) were negatively related to all assessed leaf size traits (leaf length, *L*_L_; leaf basal width, *W*_L,Base_; leaf maximum width, *W*_L,Max_; and leaf area, *A*_L_) at colony level (Fig. 6). Similar negative correlations were also found at leaf level (Supplementary Data Fig. S7).

Cyanobacterial densities of branch leaves (CD_L,Br_) and stem leaves (CD_L,St_) showed positive correlations with frequency of leaves (*F*_L_; branch leaves, *R*^2^ = 0.77, *P* = 0.002; stem leaves, *R*^2^ = 0.36, *P* = 0.041, Fig. 6). Shoot-level analyses revealed similar positive relations between frequency of leaves (*F*_L_) and cyanobacterial colonization (CC_L,Br_, CD_L,St,_ CD_L,Br_ and F_L,Colonized_, Supplementary Data Fig. S5).

### Cyanobacterial colonization as affected by pH and phenol content

**Fig. 7:**
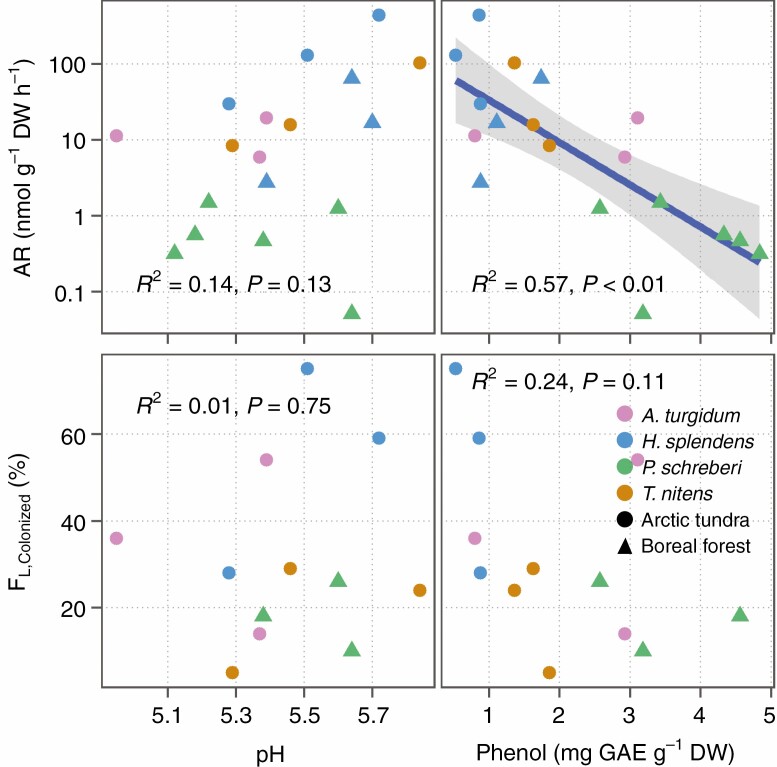
Acetylene reduction rate (AR, nmol g dw^−1^ h^−1^) and frequency of cyanobacteria-colonized leaves (*F*_L,Colonized_) in relation to pH and total phenol content of moss colonies. Each coloured dot represents one moss colony grouped by species identity indicated with different colours and site indicated with shapes. The grey shading around the regression line represent 95 % confidence interval of the fitted values.

There was no significant correlation between pH with either N_2_ fixation activity or frequency of colonized leaves (*F*_L,Colonized_). N_2_ fixation activity was negatively related to total phenol content of mosses (*R*^2^ = 0.57, *P* < 0.001, Fig. 7), while there was no significant correlation between phenol content and colonization (*F*_L,Colonized_).

### *Ecosystem differences in* H. splendens *colonies and colonization rates of paraphyllia*

**Fig. 8:**
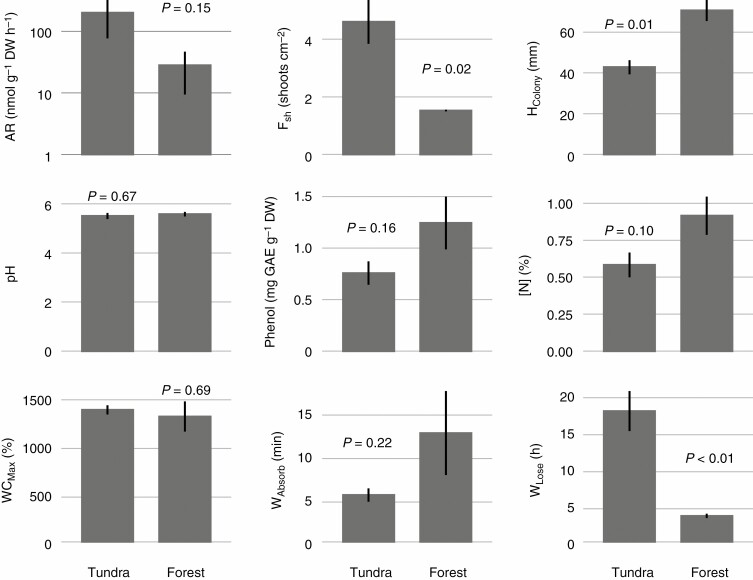
Differences in acetylene reduction rate (AR, nmol g DW^−1^ h^−1^), colony structure (*F*_Sh_, frequency of shoots and *H*_Colony_, colony height), pH, phenol content, nitrogen content ([N]) and water balance-related traits (WC_Max_, maximum water content, *W*_Absorb_, time for 50 % hydration and *W*_Lose_, time for 50 % desiccation) of *H. splendens* between the two sampled sites (arctic tundra and boreal forest).

No significant differences were found in N_2_ fixation activities in *H. splendens* between the two ecosystems (arctic tundra versus boreal forest), although the mean N_2_ fixation activity of *H. splendens* colonies from the tundra site was over 7 times higher than that of *H. splendens* colonies from the forest site (Fig. 8). However, *H. splendens* colonies from the tundra site were characterized by a higher shoot frequency (F_Sh_, *P* = 0.016), lower colony height (*H*_Colony_, *P* = 0.015) and lower desiccation rate (longer time for 50 % desiccation, *W*_Lose_, *P* = 0.006, Fig. 8) than colonies from the forest site.

**Fig. 9:**
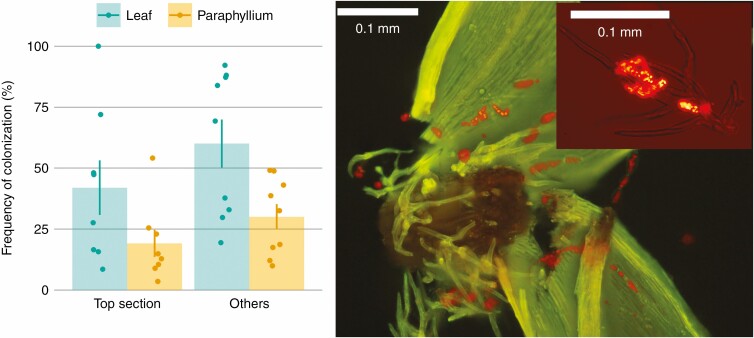
Cyanobacteria colonization (%) on leaves and paraphyllia of *H. splendens*. The left figure compares the frequency of colonized leaves with the frequency of colonized paraphyllia. Each coloured dot represents a respective section from one *H. splendens* shoot; bars show the mean ± s.e. (*n* = 9). Frequency of cyanobacterial colonization was significantly higher on leaves than on paraphyllium (*P* = 0.003, two-way ANOVA). The picture on the right shows cyanobacteria (bright orange dots) on *H. splendens* leaves and paraphyllia, and the picture in the top-right corner shows cyanobacteria attached to a paraphyllium. Photos were taken under an Olympus BX61.

Stems of *H. splendens* are covered by filamentous appendages, paraphyllia. Cyanobacteria colonized 19.2 and 30.0 % of paraphyllia on the youngest, top sections and older sections, respectively (Fig. 9). The frequency of paraphyllia colonized by cyanobacteria was significantly lower than that of leaves on the respective shoot sections (*F*_1,31_ = 10.47, *P* = 0.003).

## DISCUSSION

This study presents the first demonstration of the linkage between cyanobacterial colonization and traits of the moss hosts, while the moss could be simply used as a substrate by colonizing cyanobacteria. Further, our data supports our first hypothesis (H1), stating that N_2_ fixation activity in moss–cyanobacteria associations is closely related to the abundance of epiphytic cyanobacteria. A strong link between cyanobacterial abundance and N_2_ fixation activity has been found in previous studies (e.g. Smith, 1984; Rousk *et al*., 2013*a*). This allows an interchangeable use of activity and abundance as well as extrapolation from one to the other, easing large-scale experiments.

### Linking hydration rate and cyanobacterial colonization

Our results reveal that hydration rate of the moss host, not water content *per se*, controls cyanobacterial colonization (Fig. 4), corroborating our second hypothesis (H2), that the moss species with the highest hydration rate hosts the most cyanobacteria. Cyanobacteria can only move in liquid or on moist surfaces (Brahamsha and Bhaya, 2014) and their mobility is transient (Bay *et al*., 2013). Hence, higher hydration rates could promote cyanobacterial colonization of moss leaves via passive transport along the moss shoot. This notion could also explain Bay *et al*. (2013)’s finding that *Polytrichum commune* induces hormogonia, the cyanobacterial infectious units, but does not host cyanobacteria. This could be attributed to the endohydric nature of the moss in which, compared with ectohydric mosses, water is conducted internally and the surface of the moss is water-repellent (Proctor, 2000), thereby preventing cyanobacterial colonization via external water flow along the moss shoot. Hence, our findings could explain moisture’s positive effect on N_2_ fixation in moss–cyanobacteria associations found in earlier studies (e.g. Gundale *et al*., 2012; Rousk *et al*., 2018).

Our results that moss colonies comprising longer shoots and with wider leaves take more time to reach 50 % hydration (Fig. 5) confirmed the morphological control over hydration of moss colonies (Larson, 1981). We found morphological traits (shoot length and leaf width) related negatively to cyanobacterial colonization, and conversely, traits that facilitate hydration increase cyanobacterial colonization, supporting our third hypothesis (H3). Shoot length was negatively correlated with cyanobacterial count and density (Supplementary Data Fig. S5). This could be the result of lower hydration rates of colonies comprising longer shoots, or the longer distances the cyanobacteria need to move to colonize longer shoots, or a combination of both. Confirming the shoot length effect, we found a lower frequency of colonized leaves at the top 1- to 2-cm section than that for other sections (Supplementary Data Fig. S6). Leaf width was negatively correlated with hydration rate, and thus negatively correlated with colonization (Fig. 5). Although wider leaves were found to hold more water by enabling leaves to curve in *Sphagnum* species (Såstad and Flatberg, 1993; Bengtsson *et al*., 2020), the increase in leaf width results in a lower number of leaves per unit shoot length (Niinemets and Tobias, 2019), which reduces water-holding capacity and hydration rate at the shoot level.

### Leaf size and number trade-off, and the effect on cyanobacterial colonization

Surprisingly, all measured traits related to leaf size (area, width etc.) were negatively correlated with cyanobacterial colonization, probably related to desiccation rates of larger, broader leaves, while leaf frequency was positively related to colonization (Fig. 6), supporting our fourth hypothesis (H4), that moss shoots with higher frequency of leaves host more cyanobacteria.

The negative relationship between leaf size and colonization is likely driven by a trade-off between leaf size and number. Leaf size is negatively correlated to leaf number in both vascular plants (Kleiman and Aarssen, 2007) and mosses (Niinemets and Tobias, 2019). We found a strong negative correlation between leaf frequency (*F*_L_) and area (*A*_L_) (Supplementary Data Fig. S4), confirming the leaf size versus number trade-off. The trade-off implies that the smaller the leaf size, the more leaves per unit stem length the plant can carry. Mosses with up to 55 leaves mm^−1^ shoot length fall at the extreme end of the size versus number trade-off (Niinemets and Tobias, 2019). In *H. splendens* collected from the tundra, we found a range of 52–115 leaves mm^−1^ shoot, which is much higher than leaf frequencies from the study above, and an area of 0.23 ± 0.01 mm^2^ for branch leaves and 0.48 ± 0.04 mm^2^ for stem leaves. In correspondence to the extremely high leaf frequency, *H. splendens* hosts the most cyanobacteria among the studied mosses (Fig. 6), and has the highest N_2_ fixation activity (Figs 2 and 3). On the other end of the trade-off spectrum, *P. schreberi* has the largest leaves, with an area of 0.70 ± 0.05 mm^2^ for branch leaves and 1.41 ± 0.07 mm^2^ for stem leaves, and a low leaf frequency (33.90 ± 3.78 leaves mm^−1^ shoot) among the three pleurocarpous mosses. *Pleurozium schreberi* was the least colonized by cyanobacteria and had the lowest N_2_ fixation activity. *Tomentypnum nitens* has slightly smaller leaves (0.64 ± 0.02 mm^2^ for branch leaves and 1.26 ± 0.09 mm^2^ for stem leaves) than *P. schreberi* and a similar leaf frequency of 33.35 ± 4.24 leaves mm^−1^ shoot length. Accordingly, *T. nitens* has a slightly higher cyanobacterial colonization rate than *P. schreberi*. Although the non-branching acrocarp, *A. turgidum*, has the largest leaf area (1.68 ± 0.07 mm^2^) and lowest leaf frequency (9.12 ± 0.98 leaves mm^−1^ shoot length) among the studied mosses, colonization rate and N_2_ fixation activity was still higher than that of *P. schreberi*, probably because water balance of non-branching acrocarps depend primarily on shoot density (Niinemets and Tobias, 2019). Species with an extremely high frequency of small leaves have shoots with large surface area, which increase water absorption (Larson, 1981), and provide more potential colonization sites for cyanobacteria than those mosses with fewer, larger leaves.

### Phenol content and pH effects on cyanobacterial colonization

In our experiment, phenols affected cyanobacterial activity negatively, but not cyanobacterial colonization. This fits with previous findings showing that moss phenol content did not correlate with cyanobacterial colonization (Rousk *et al*., 2013*a*). The cyanobacterial infective units, hormogonia, are of higher tolerance than other cyanobacterial cells and could be less affected by phenols while colonizing (Damerval *et al*., 1991), but once differentiated to N_2_-fixing heterocytes, phenols can inhibit N_2_ fixation activity. However, a study of cycad–cyanobacteria symbiosis suggested that phenols provide a mechanism for excluding other microbes and permitting cyanobacteria to grow in cycad roots (Obukowicz *et al*., 1981). Further study is needed to disentangle the effects of phenolics and other secondary compounds on moss and cyanobacteria associations.

The studied mosses, with pH ranges from 5.0 to 6.0, might limit the activity of cyanobacteria, since the optimum pH for N_2_ fixation in mosses is between 5.9 and 6.2 (Smith, 1984). However, neither N_2_ fixation nor colonization was significantly affected by pH in our study. The pH of the moss with the highest cyanobacterial colonization and activity, *H. splendens*, ranged between 5.28 and 5.72, which is similar to the pH of the least colonized moss, *P. schreberi*, whose pH ranged between 5.32 and 5.64. This suggests that pH is not the most important determinant of cyanobacterial colonization and activity given the moss pH does not drop below 5.

### The role of ecosystem types

We note that a potential weakness of our trait analyses is that they included both inter- and intraspecific variation. We argue that trait correlations were mainly driven by interspecific variations. Samples of each species were harvested in the same habitat, and intraspecific trait variations, which are attributed to environmental factors should be small (Roos *et al*., 2019). The analyses of CVs of traits confirmed that intraspecific variations of traits were generally smaller than interspecific variations (Supplementary Data Table S1). For instance, the intraspecific CV of *P. schreberi* leaf frequency is significantly smaller than interspecific CV (0.12 versus 0.82, *P* = 0.04, modified signed-likelihood ratio test). Moreover, to demonstrate the intraspecific variation of traits and N_2_ fixation rate, we compared several traits of *H. splendens* between ecosystem types (subarctic tundra versus boreal forest). *Hylocomium splendens* colonies from the tundra were shorter and had more shoots per area than the colonies from the forest (Fig. 7). These differences between ecosystems are likely driven by environmental factors, such as temperature, moisture (Bisbee *et al*., 2001) and nutrient availability, as the tundra has a lower mean annual temperature (0.2 versus 1 °C) and precipitation (337 versus 570 mm) than the forest site (Rousk *et al*., 2014; Rousk and Michelsen, 2017), impacting moss growth. Shoot size controls water fluxes in moss colonies (Elumeeva *et al*., 2011) (see section: Linking hydration rate and cyanobacterial colonization), and can thereby control cyanobacterial colonization. Fine-scale variations in colony structure, such as changes in shoot frequency, alter surface roughness and further interact with wind flow affecting boundary-layer properties and desiccation rate (Rice and Schneider, 2004; Michel *et al*., 2012). This is confirmed by the distinct desiccation rate for *H. splendens* colony between tundra and forest sites (Fig. 7). It is possible that trait variation, driven by ecosystem specific factors (e.g. N availability, temperature), are responsible for intraspecific differences in cyanobacterial colonization and thus N_2_ fixation, while relative interspecific variation should remain similar in ecosystems with different abiotic conditions.

We did not assess cyanobacterial colonization for *H. splendens* from the forest site, but given that N_2_ fixation activity is closely linked to cyanobacterial colonization, and activity was not different between the sites, it is likely that colonization is similar between the sites, too (Fig. 8). Moreover, in contrast with *H. splendens* collected from the forest site, *P. schreberi* had still lower N_2_ fixation activity (27.75 versus 0.68 nmol g^−1^ DW h^−1^, Fig. 3). Hence, the variation in cyanobacterial colonization and, thus N_2_ fixation activity, should be largely controlled by species-specific traits, but intraspecific variation that affects colonization is also in turn driven by the environment.

### *Morphological peculiarity of* H. splendens

Our results present the first evidence that cyanobacterial colonization of paraphyllia can be substantial. About 20–30 % of paraphyllia were colonized by cyanobacteria (Fig. 9). Given the large number of paraphyllia along the moss shoot, associated cyanobacteria can account for a considerable portion of N_2_ fixation. Hence, focusing on moss leaves as colonization sites could vastly underestimate cyanobacterial colonization along moss shoots. Paraphyllia occur in taxa of only a few moss families, such as Hylocomiaceae, Thuidiaceae and Brachytheciaceae (Spirina *et al*., 2020). If those taxa that have paraphyllia host more cyanobacteria than taxa without these structures is an open question. Moreover, paraphyllia, which are photosynthetic filaments or leaf-like structures composed of live cells, are located between leaf and stem, where many N_2_-fixing cyanobacteria occur. Although these thick-walled cells may have low nutrient exchange rates, it is possible that paraphyllia could promote N uptake by the moss host by creating a link between epiphytic cyanobacteria and the moss stem. These open questions and unknowns call for further research on the paraphyllium’s role in the mosses’ N uptake, which could act as a lens through which to resolve the link between traits and ecological functions.

In conclusion, our findings emphasize that the hydration rate of a moss colony is a key trait regulating cyanobacterial colonization. On the other hand, species-specific morphological traits control hydration rate. Chemical traits of the moss host seem to be less important than morphological traits in regulating cyanobacterial colonization. We could also demonstrate that a considerable portion of cyanobacteria colonize paraphyllia, a previously overlooked structure in terms of cyanobacterial colonization. Variation in moss species-specific traits drives cyanobacterial colonization, but intraspecific variation that affects colonization is in turn driven by environmental factors. Given that mosses are a key source of N to ecosystems where they dominate the ground cover, uncovering the relation between moss traits and cyanobacterial colonization will ultimately result in a better estimation of the amount of N input – as dependent on moss traits – and can provide new perspectives and information for understanding the relationship between mosses and cyanobacteria.

## SUPPLEMENTARY DATA

Supplementary data are available online at https://academic.oup.com/aob and consist of the following. Figure S1: pictures of the studied moss species. Figure S2: change in moss colony weight over time for four moss species during water absorption and loss. Figure S3: acetylene reduction rate in relation to the cyanobacteria count and density on moss leaves. Figure S4: relationship between leaf area and frequency of leaves for individual moss shoots. Figure S5: relationships between cyanobacterial colonization and shoot traits. Figure S6: differences in frequency of colonized leaves between top segments and lower segments. Figure S7: relationships between cyanobacterial colonization and leaf size. Table S1: intra- and interspecific CVs of water balance, colony, chemical and morphological traits.

mcab127_suppl_Supplementary_Figure_S1

mcab127_suppl_Supplementary_Materials_S1

mcab127_suppl_Supplementary_Materials_S2
