# Supplementary figures and images for: The moss traits that rule cyanobacterial colonization
Source: Ann Bot. 2021 Oct 10;129(2):147–60. doi: 10.1093/aob/mcab127 (PMC8796673; doi:10.1093/aob/mcab127)

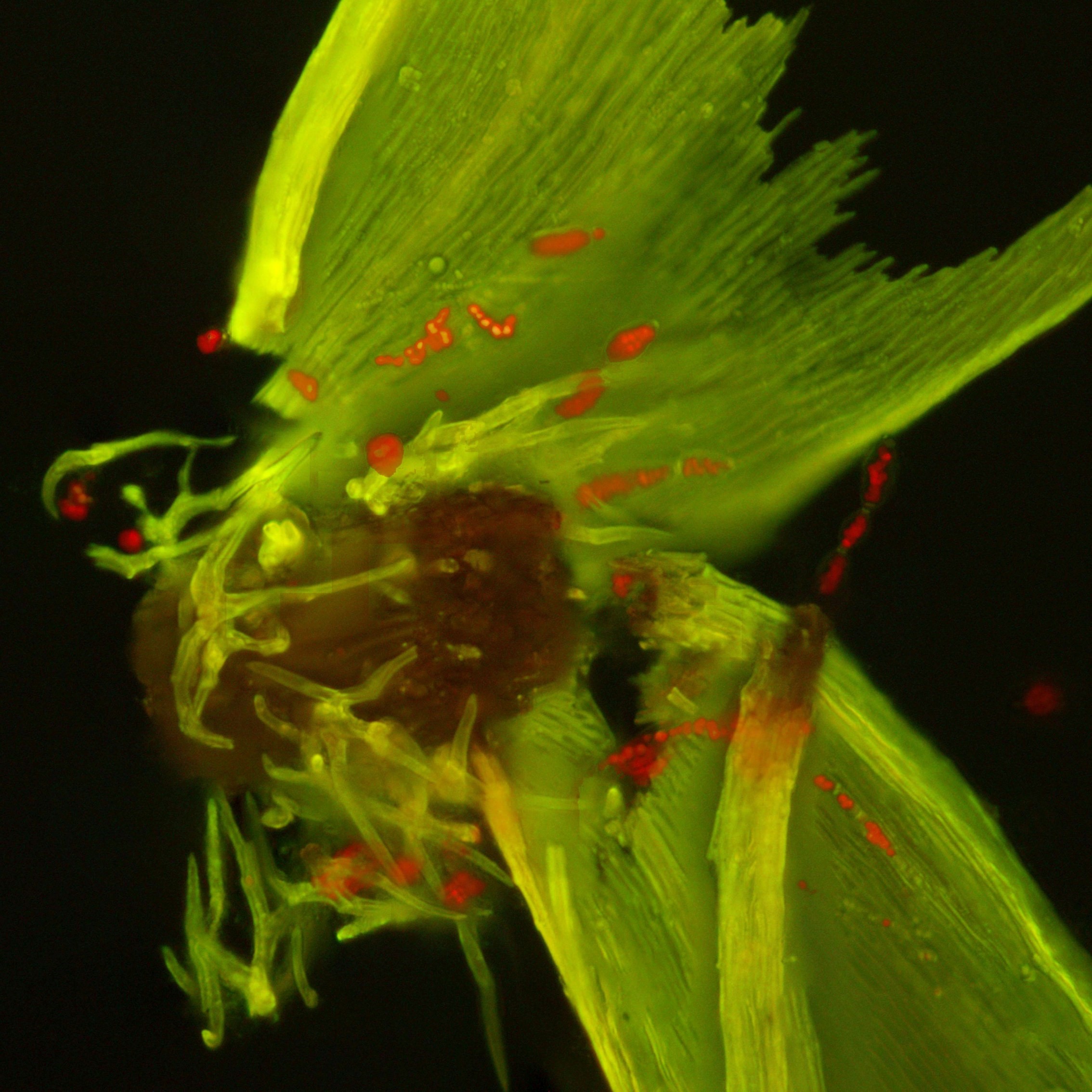

Supplement: mcab127_suppl_Supplementary_Figure_S1 [file mcab127_suppl_supplementary_figure_s1.jpeg]
