# Supplementary material for: The moss traits that rule cyanobacterial colonization
Source: Ann Bot. 2021 Oct 10;129(2):147–60. doi: 10.1093/aob/mcab127 (PMC8796673; doi:10.1093/aob/mcab127)
Supplement: mcab127_suppl_Supplementary_Materials_S1 [file mcab127_suppl_supplementary_materials_s1.doc]

The inset cover illustration shows a section of Hylocomium splendens stem with leaves and paraphyllia under an UV-fluorescence microscope. The moss associated cyanobacteria are seen in bright red. The colonization of cyanobacteria is related to water balance and morphological traits of moss host.
