# Supplementary material for: The moss traits that rule cyanobacterial colonization
Source: Ann Bot. 2021 Oct 10;129(2):147–60. doi: 10.1093/aob/mcab127 (PMC8796673; doi:10.1093/aob/mcab127)
Supplement: mcab127_suppl_Supplementary_Materials_S2 [file mcab127_suppl_supplementary_materials_s2.doc]

Supplementary Information.

Authors: Xin Liu and Kathrin Rousk.

Title: The moss traits that rule cyanobacterial colonization.

Seven figures and 1 table.

Figure S1 Pictures of the studied moss species.

Figure S2 The change of moss colony weight over time for four moss species during water absorption and loss.

Figure S3 Acetylene reduction rate in relation to the cyanobacteria count and density on moss leaves.

Figure S4 Relationship between leaf area and frequency of leaves for individual moss shoots.

Figure S5 Relationships between cyanobacterial colonization and shoot traits.

Figure S6 Differences in frequency of colonized leaves between top segments and lower segments.

Figure S7 Relationships between cyanobacterial colonization and leaf size.

Table S1 Intra- and inter-specific coefficient of variation (CV) of water balance, colony, chemical and morphological traits.


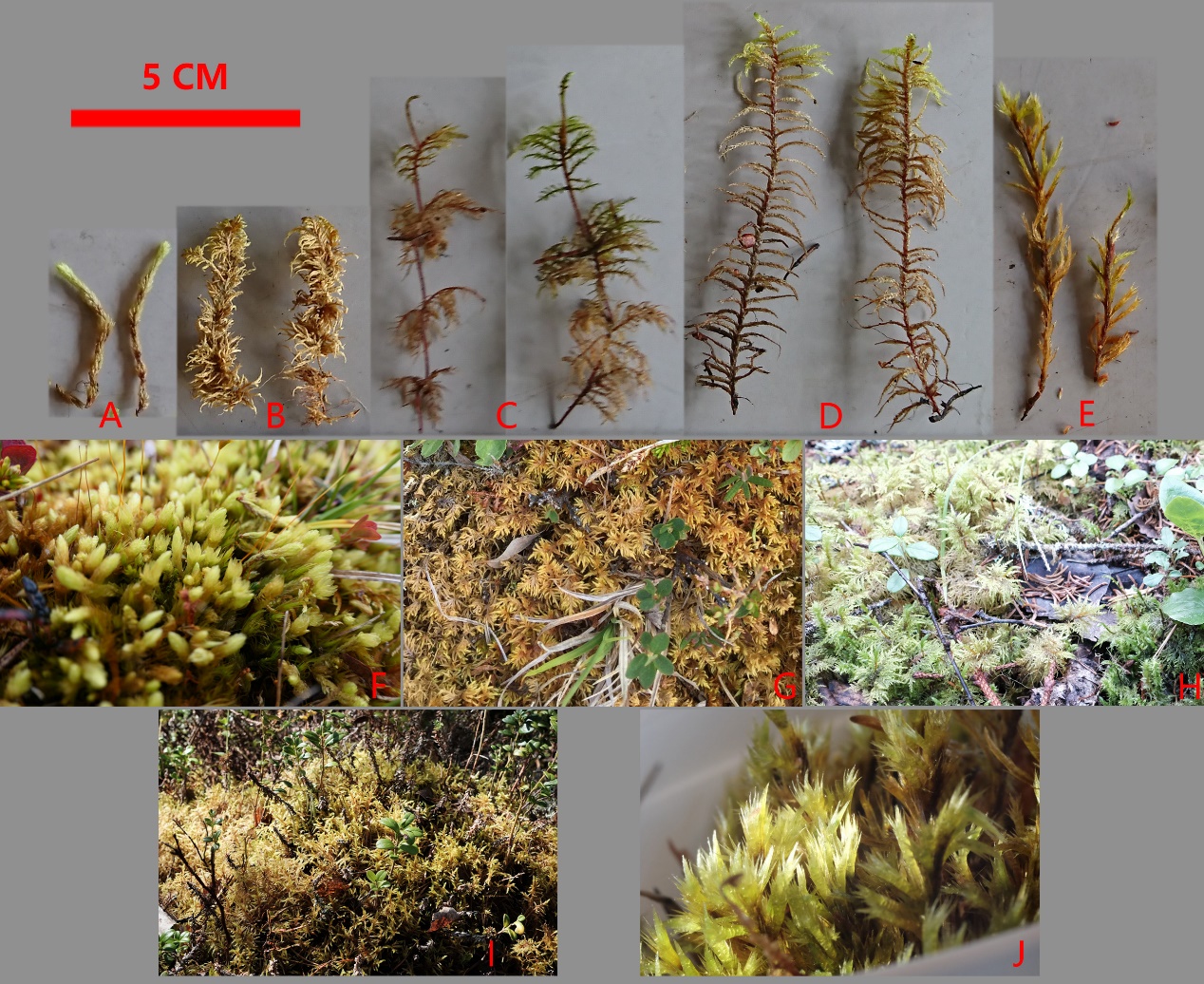


Figure S1 Pictures of the studied moss species. Shoots of *Aulacomnium turgidum* (A), *Hylocomium* *splendens* from arctic tundra (B) and boreal forest (C), *Pleurozium* *schreberi* (D), and *Tomentypnum* *nitens* (E). Colonies of *A. turgidum* (F), *H. splendens* from arctic tundra (G) and boreal forest (H), *P. schreberi* (I), and *T. nitens* (J). Pictures A-E are in proportion to the scale bar.


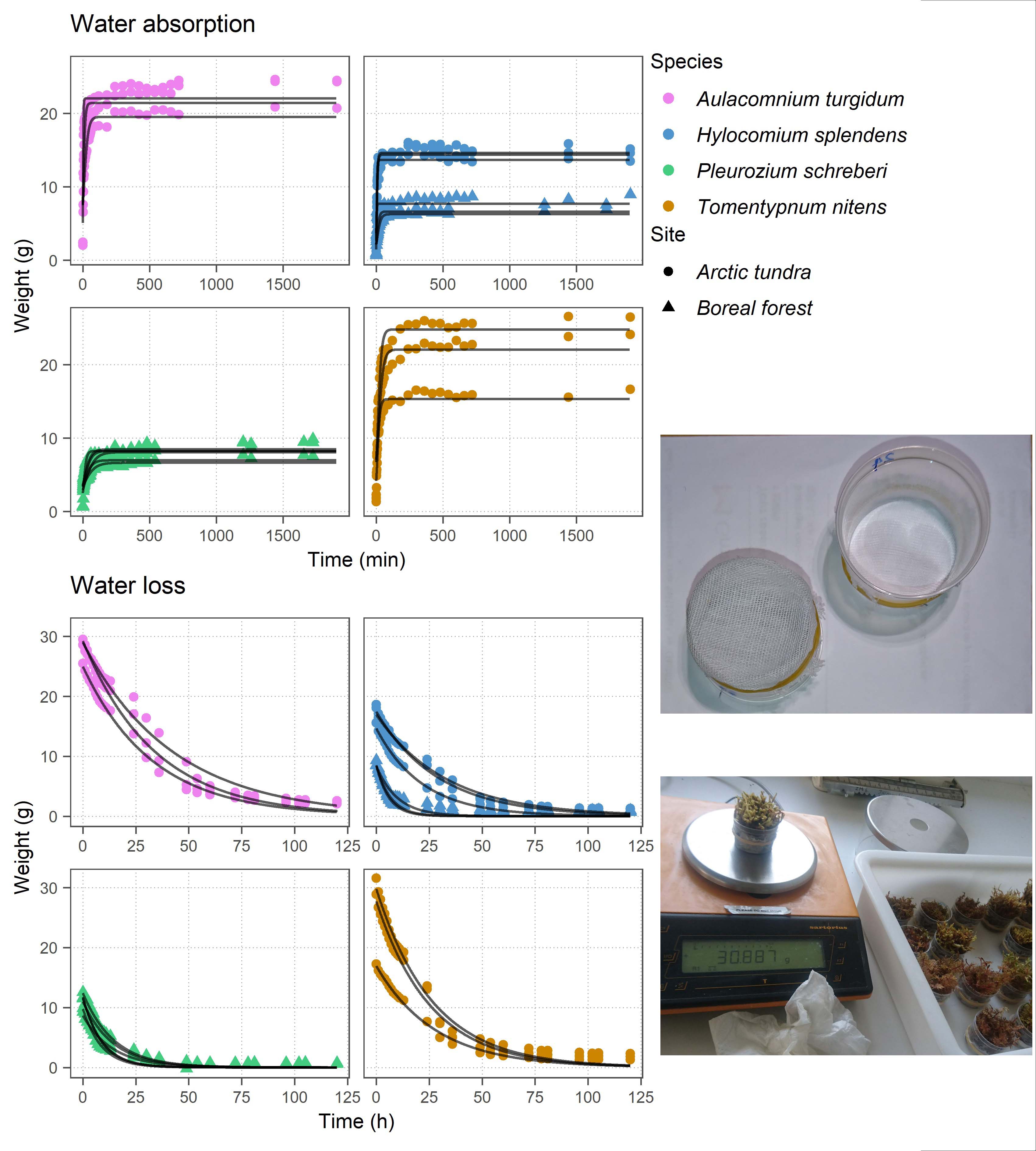


Figure S2 The change of moss colony weight over time for four moss species during water absorption and loss. Exponent curves, weight = K / (1 + exp(a+b*time)) and weight = a * exp(-b*time), were fitted to the water absorption and loss processes respectively. The pictures to the right shows the containers used and the experiment setting in the water balance measurement.


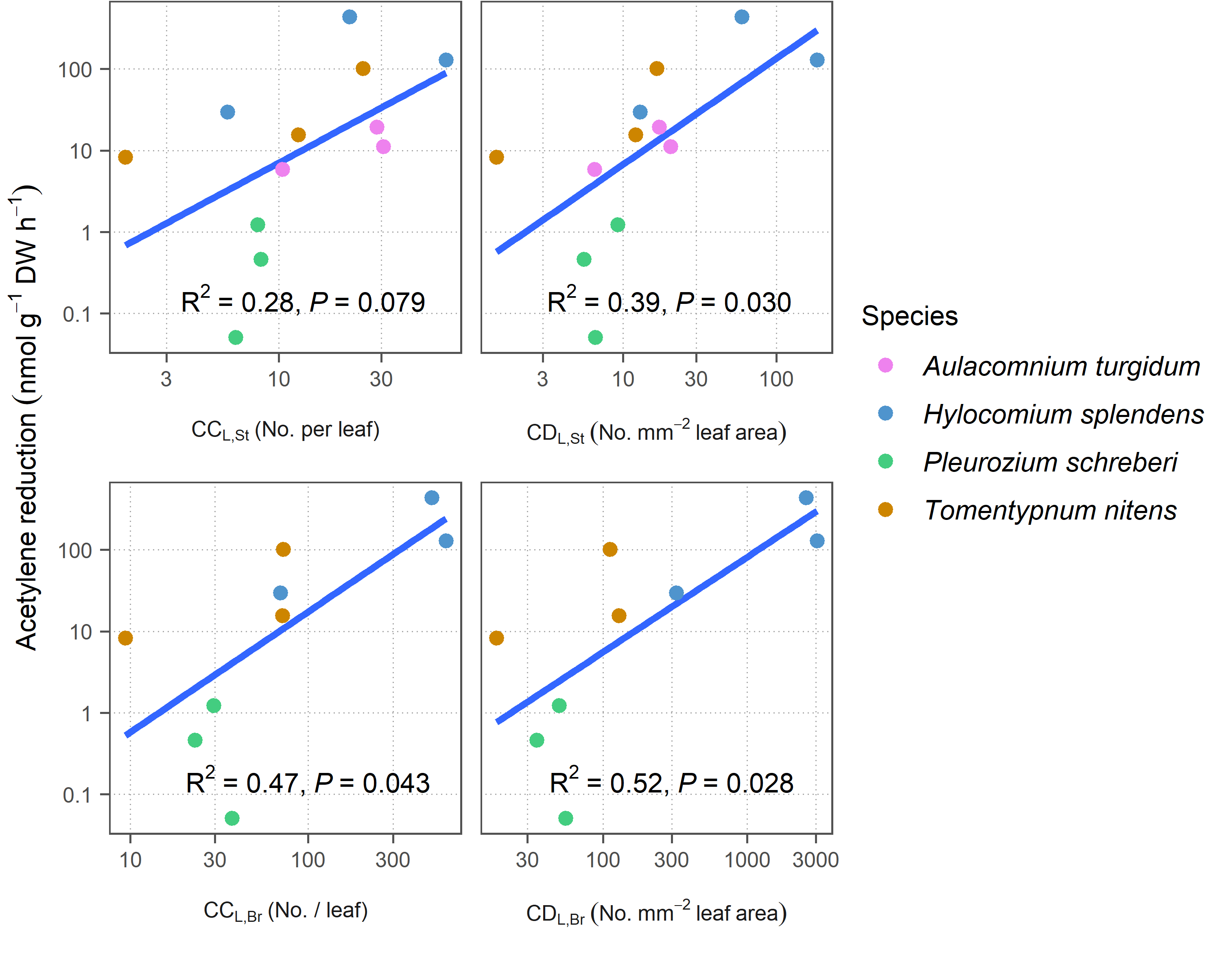


Figure S3 Acetylene reduction rate in relation to the cyanobacteria count (CC) and density (CD) on moss leaves. Each colored dot represents one moss colony which was grouped by species identity indicated with different colors. All variables was log10-transformed before analyses.


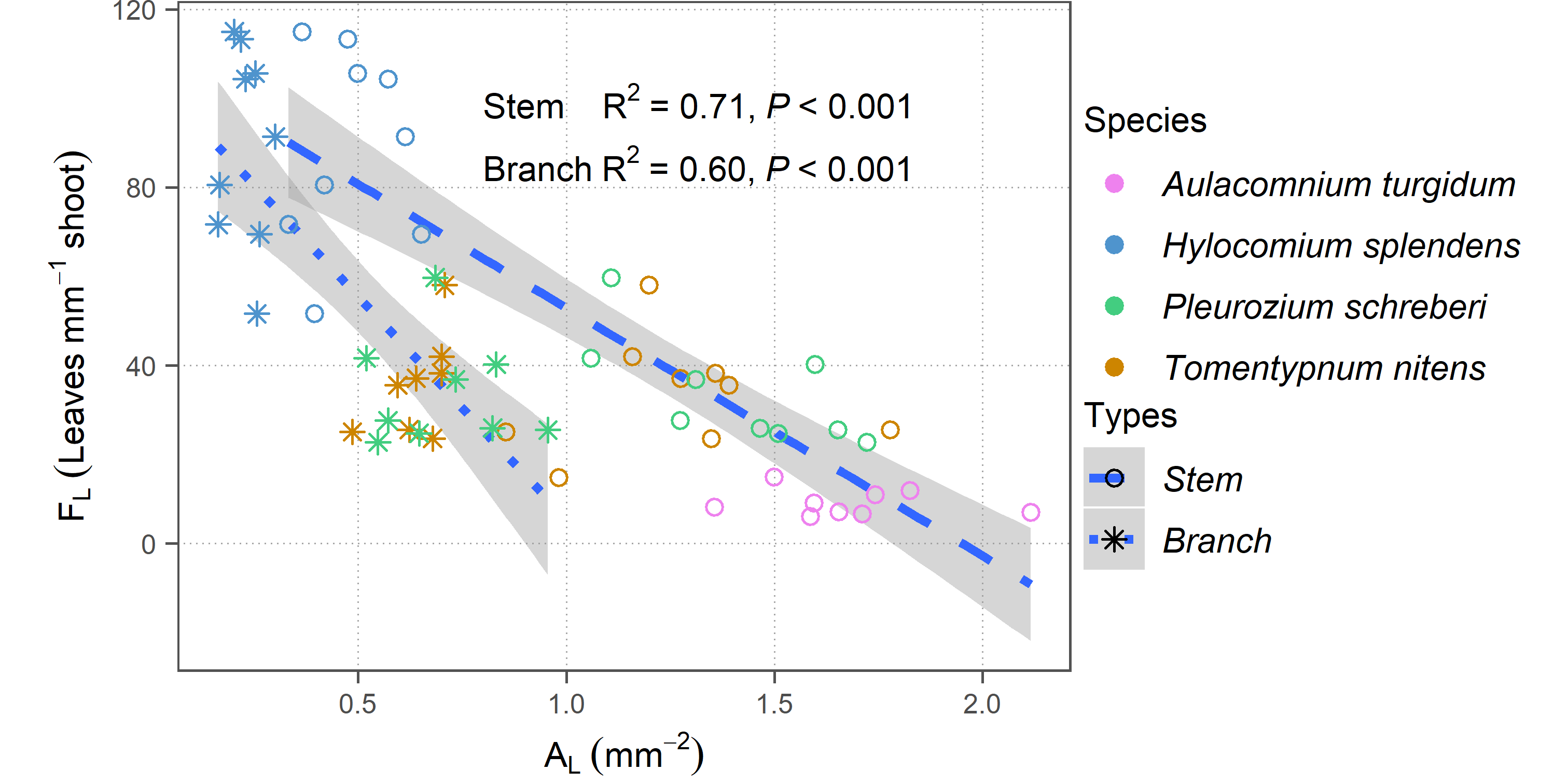


Figure S4 Relationship between leaf area (A_L_) and frequency of leaves (F_L_) for individual moss shoots. Shown are shoot level mean values of A_L_, obtained by averaging the area of 10-107 leaves. The data for area of stem leaves and branch leaves were fitted against F_L_ of individual shoots separately.


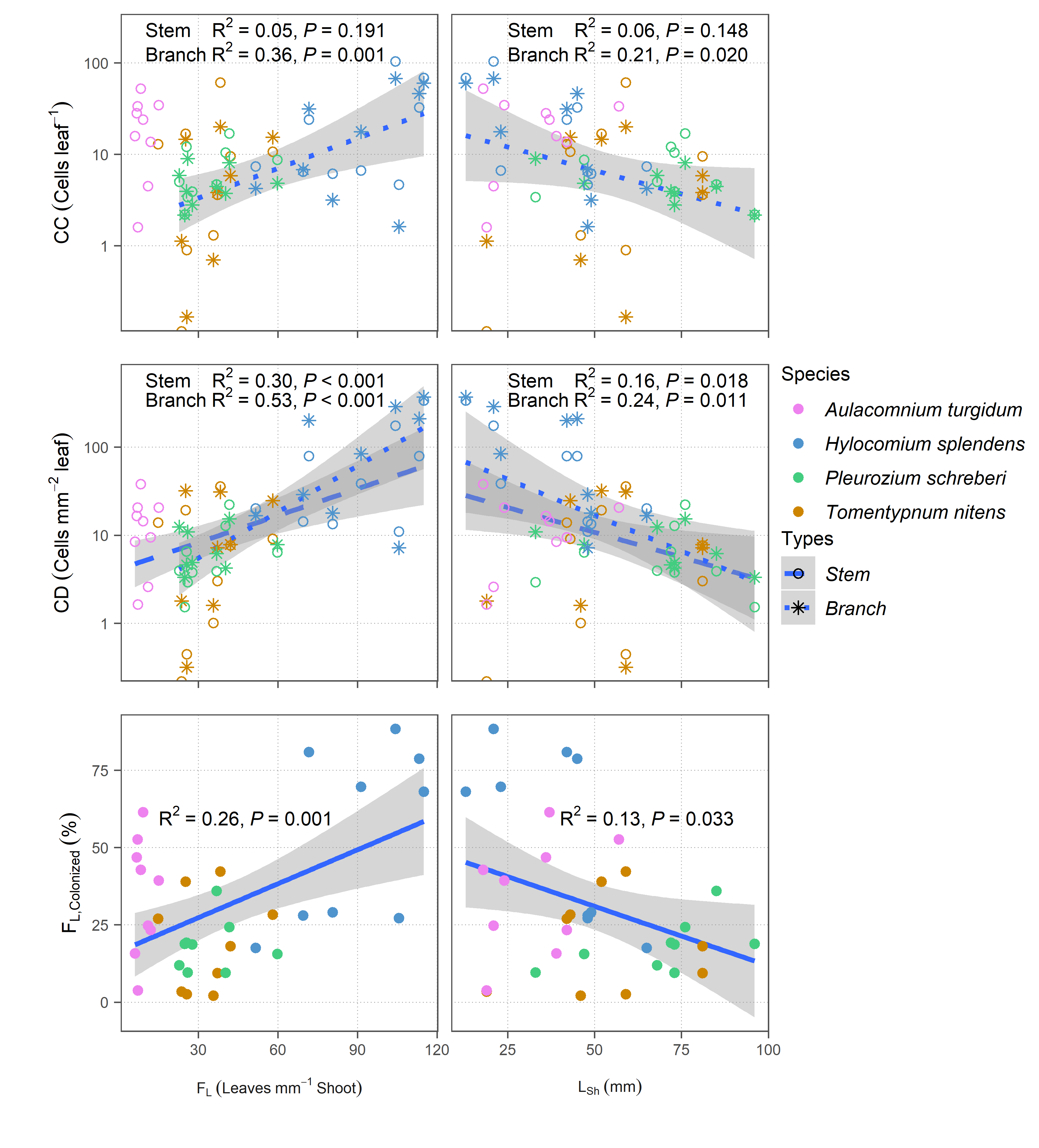


Figure S5 Relationships between cyanobacterial colonization, in terms of cyanobacteria count (CC), density (CD) and frequency of colonized leaves (F_L,Colonized_) and shoot traits, in terms of frequency of leaves (F_L_) and shoot length (L_Sh_), for individual moss shoots. Shoot level mean values for CC and CD, which were obtained by averaging these of 10-107 leaves, are shown. Cyanobacteria count and density were log10-transformed before analyses.

**
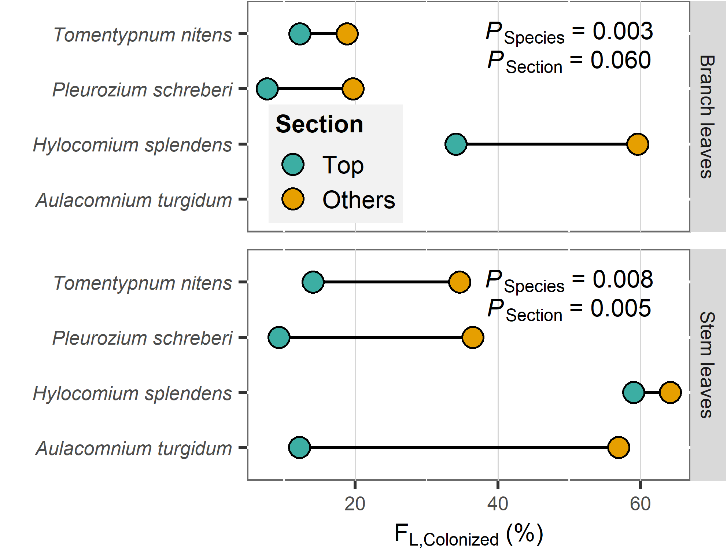
**

Figure S6 Differences in frequency of colonized leaves (F_L,Colonized_) between top segments and lower (other) segments. Each colored dot represents the mean frequency of colonized leaves of the respective section from each species, which was averaged from three replicates.


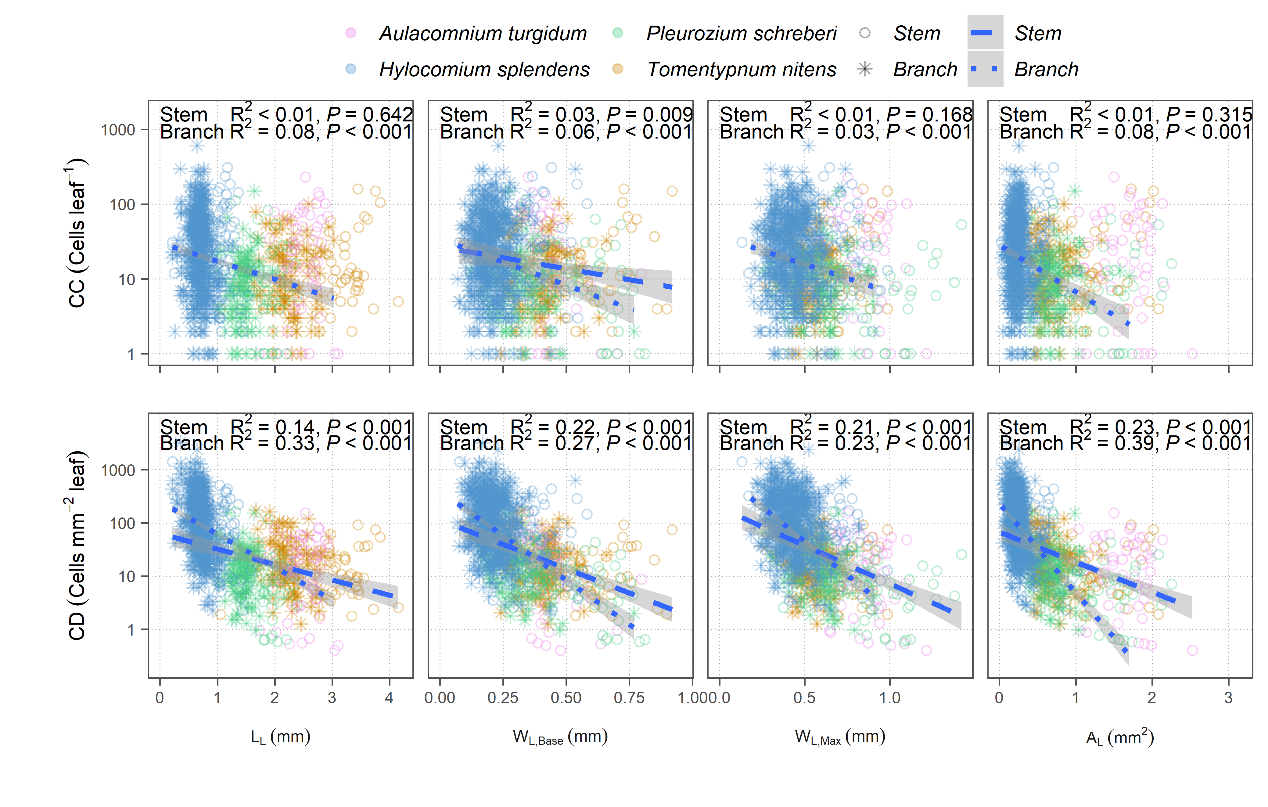


Figure S7 Relationships between cyanobacterial colonization, in terms of cyanobacterial count (CC) and density (CD) and leaf size, in terms of leaf length (L_L_), basal width (W_L,Base_), maximum width (W_L,Max_), and area (A_L_), for individual moss leaves. Cyanobacteria count and density were log10 transformed before analyses. Leaves without any epiphytic cyanobacteria were not included in the analyses.

Table S1 Intra- and inter-specific coefficient of variation (CV) of water balance, colony, chemical and morphological traits. Species mean values were used to calculate interspecific coefficient of variation. Krishnamoorthy and Lee’s (2014) modified signed-likelihood ratio test (MSLR) was used to test the differences between species CV and interspecific CV. “+” indicates P values less than 0.1, “*” indicates P values less than 0.05, and “**” indicates P value less than 0.01.

|  | WC_Max_ | W_Absorb_ | W_Lose_ | H_Colony_ | F_Sh_ | pH | Phenol | L_Sh_ | F_L_ | W_L,Base,St_ | W_L,Max,St_ | L_L,St_ | A_L,St_ |
| --- | --- | --- | --- | --- | --- | --- | --- | --- | --- | --- | --- | --- | --- |
| *Aulacomnium turgidum* | 0.03* | 0.64 | 0.14 | 0.34 | 0.05** | 0.05 | 0.56 | 0.34 | 0.22 | 0.05+ | 0.12 | 0.04* | 0.08+ |
| *Hylocomium splendens* | 0.13 | 0.71 | 0.75 | 0.3 | 0.61 | 0.03 | 0.41 | 0.45 | 0.14+ | 0.07+ | 0.07 | 0.08+ | 0.14 |
| *Pleurozium schreberi* | 0.09 | 0.29 | 0.24 | 0.18 | 0.16* | 0.04 | 0.23 | 0.21 | 0.12* | 0.09 | 0.05+ | 0.09+ | 0.11 |
| *Tomentypnum nitens* | 0.08 | 0.26 | 0.06* | 0.22 | 0.24 | 0.05 | 0.15 | 0.22 | 0.24 | 0.14 | 0.07 | 0.07* | 0.16 |
| Inter-species | 0.2 | 0.59 | 0.52 | 0.26 | 0.61 | 0.03 | 0.56 | 0.33 | 0.82 | 0.27 | 0.21 | 0.42 | 0.43 |
